# Correlative chemical and elemental nano-imaging of morphology and disorder at the nacre-prismatic region interface in *Pinctada margaritifera*

**DOI:** 10.1038/s41598-023-47446-5

**Published:** 2023-12-01

**Authors:** Brian T. O’Callahan, Amy Larsen, Sarah Leichty, John Cliff, Alex C. Gagnon, Markus B. Raschke

**Affiliations:** 1 Environmental and Molecular Sciences Division, Pacific Northwest National Laboratory, Richland, WA USA; 2 School of Oceanography, University of Washington, Seattle, WA USA; 3 Department of Physics, and JILA, University of Colorado at Boulder, Boulder, CO USA

**Keywords:** Element cycles, Imaging techniques, Structural biology, Nanoscience and technology

## Abstract

Understanding biomineralization relies on imaging chemically heterogeneous organic–inorganic interfaces across a hierarchy of spatial scales. Further, organic minority phases are often responsible for emergent inorganic structures from the atomic arrangement of different polymorphs, to nano- and micrometer crystal dimensions, up to meter size mollusk shells. The desired simultaneous chemical and elemental imaging to identify sparse organic moieties across a large field-of-view with nanometer spatial resolution has not yet been achieved. Here, we combine nanoscale secondary ion mass spectroscopy (NanoSIMS) with spectroscopic IR *s*-SNOM imaging for simultaneous chemical, molecular, and elemental nanoimaging. At the example of *Pinctada margaritifera* mollusk shells we identify and resolve ~ 50 nm interlamellar protein sheets periodically arranged in regular ~ 600 nm intervals. The striations typically appear ~ 15 µm from the nacre-prism boundary at the interface between disordered neonacre to mature nacre. Using the polymorph distinctive IR-vibrational carbonate resonance, the nacre and prismatic regions are consistently identified as aragonite (${\overline{\nu }}_{a}=860$ cm^−1^) and calcite (${\overline{\nu }}_{c}=880$ cm^−1^), respectively. We observe previously unreported morphological features including aragonite subdomains encapsulated in extensions of the prism-covering organic membrane and regions of irregular nacre tablet formation coincident with dispersed organics. We also identify a ~ 200 nm region in the incipient nacre region with less well-defined crystal structure and integrated organics. These results show with the identification of the interlamellar protein layer how correlative nano-IR chemical and NanoSIMS elemental imaging can help distinguish different models proposed for shell growth in particular, and how organic function may relate to inorganic structure in other biomineralized systems in general.

## Introduction

Biological structures are often characterized patterns that are self-similar, fractal, or periodic, over a hierarchy of length scales serving specific metabolic, skeletal, locomotory, and other functions^1–5^. In addition, many of these structural motifs have inspired numerous man-made engineering designs including photonic devices^6^, lightweight aerospace materials based on avian bone structure^1,7^, increased material strength based on biomineralization^8^, or reduced hydrodynamic drag by emulating shark skin^9^. Further, functional biological structural motives can serve as inspiration to address societal challenges including carbon sequestration^10^, and climate change research^11^ as well as numerous medical applications including bone implants^12^, and dental remineralization^13^.

One of the fascinating phenomena in biomineralization is how the growth of even large scale functional structures (bones, teeth, shells, corals, etc.) is often facilitated, directed, and controlled by moieties of active organic layers and conduits controlling flow and precipitation of the silicate, carbonate, or phosphate mineralization^1,8^.

In that regard the shells of mollusks are particularly fascinating, with a wide range of shapes and forms, serving a variety of functions of passive protection, active defense, locomotion, adhesion, and nutrition^3,4^. However, despite intense research and many models proposed to explain the mechanism of biomineralization^14–19^, specifically relating how the active organic moiety phases control the development of the inorganic biomineralized phase has remained a major challenge.

The precipitation of structurally supporting inorganic materials via organic phases is characterized by heterogeneities on a hierarchy of length scales ranging from nanoscale aragonite tablets in the nacre to mm scale organization of calcite prisms. Probing the heterogeneity and degree of order and disorder can yield valuable insight into the elementary steps and reaction mechanisms of the biomineralization process including the role of organics in the transformation of amorphous to well-defined crystalline phases of specific polymorphs. For their understanding, a multiscale imaging technique is needed which can capture both the nanoscale localization of organics within the large-scale carbonate matrix, as well as the variations and spatial arrangement across the 10’s of micrometers over which the shell growth pattern is established.

**Figure 1:**
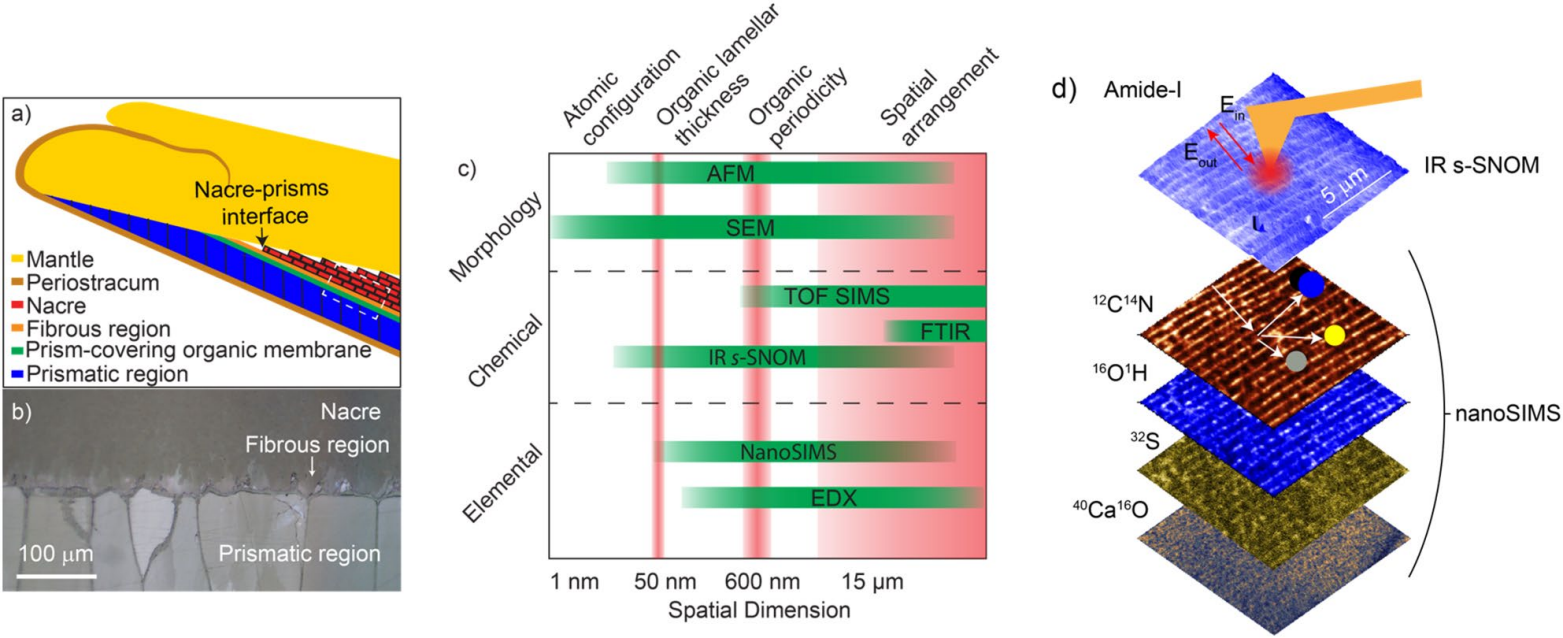
Overview of experimental design (**a**) Schematic of *P. margaritifera* shell structure and composition at the interface between the nacre and the prismatic region. (**b**) Optical image of the nacre-prisms interfacial region indicated by the box in a). (**c**) A summary of select existing technologies applied to biomineralization imaging arranged by spatial resolution and specificity. (**d**) Experimental concept. Combined IR *s*-SNOM and NanoSIMS gives correlated chemical and elemental information with nanoscale spatial resolution.

*Pinctada margaritifera,* a bivalve mollusk, is a model system at the focus of many prior imaging and chemical analyses aimed at identifying the biomolecular mechanisms controlling its shell growth^20^. Figure 1a) schematically shows the composition and texture of the shell of *P. Margaritifera*. The prismatic region precipitates onto the periostracum forming organic-encapsulated calcite subregions approximately 50–200 μm wide and up to a few millimeters in height^20,21^. Nacre is then deposited on the few-micrometer thick prism-covering organic membrane. Electron microscopy (EM) near the nacre-prismatic region interface (NPI) has identified a ~ 10–20 μm thick disordered ‘fibrous aragonite’ (FA) region upon which the regular few micrometer long, ~ 500–600 nm thick mature nacre tablets appear, separated by sub-100 nm thick interlamellar membranes (Fig. 1b)^14,22–24^. Time-of-flight secondary ion mass spectroscopy (TOF–SIMS) imaging identified and mapped the amino acids and phospholipids near the NPI with few micrometer spatial resolution^23^. With superior spatial resolution, NanoSIMS has identified organic carbon and nitrogen within the interlamellar membrane and imaged the loss of order in the fibrous aragonite region^22^, and although it provides isotopic information, lacks the ability to provide the molecular identity of the organic phases. Micro-Fourier-transform infrared (FTIR) microscopy could provide these chemical maps and track the calcium carbonate polymorph across 10’s micrometer field of view^14^, but without sufficient spatial resolution to localize the nanoscale organic layers or to assess nanoscale variations in the carbonate structure. Therefore, *no technique has yet assessed the order/disorder with simultaneous nanoscale spatial resolution and 10’s of micrometer field of view with sufficient chemical sensitivity.*

Figure 1c highlights this experimental challenge comparing the range and limitations of different experimental techniques that can map and chemically characterize biomineralized systems. Electron microscopy (EM) and atomic force microscopy (AFM) can provide insight into nacre tablet and calcite prism formation through nanometer spatially resolved mapping of sample morphology^22–25^, yet they lack chemical or elemental information. Electron dispersive X-ray spectroscopy (EDX) can image elemental composition with high spatial resolution, yet cannot distinguish organic from inorganic carbon. FTIR provides the desired chemical information yet lacks sufficient spatial resolution. Chemical imaging with TOF–SIMS can afford improved spatial resolution however is typically limited to features down to ~ 1 μm in dimension^23^.

In contrast, infrared scattering-scanning near-field optical microscopy (IR *s*-SNOM) and NanoSIMS, the nanoscale extensions of FTIR and SIMS, provide the chemical specificity of infrared spectroscopy and the elemental analysis of mass spectroscopy, respectively, with nanometer spatial resolution and high sensitivity. In this work, we use IR *s*-SNOM to identify the chemical nature of the organic carbon as collocated in NanoSIMS and correlate it with ionic composition (Ca^2+^, Mg^2+^), as well as the type of carbonate polymorph (Fig. 1d). With the example of *P. margaritifera* we identify and map the chemical shell content near the interface between the nacre and prismatic regions and identify regions of order and disorder across 10’s of micrometer field of view to capture the growth characteristics of the first layers of nacre. Using IR *s*-SNOM we nano-image the calcium carbonate polymorphs and identify that the morphology of the calcite varies near the calcite-aragonite interface, yet the polymorphs are clearly separated by the prism-covering membrane^14^. We also map incipient nacre formation where FA transitions to regular tablets separated by interlamellar organic sheets. Prior NanoSIMS work has observed organic carbon and nitrogen in these organic sheets, and our chemical nano-imaging detects amide I in the ~ 50 nm thick sheets. These layers typically appear ~ 15 µm from the nacre-prism boundary at the interface between disordered neonacre and mature nacre, however in some locations less well-defined tablets appear along with dispersed organics closer to the prismatic region indicating variability in the growth process.

The results show the utility of IR *s*-SNOM to assess biomineralization processes on the nanometer scale, identify the nature of the organic material (lipid, protein, saccharide, etc.), and combined with NanoSIMS associate inorganic mineralization products with their organic crystallization catalysts in real space, which we expect can aid in the refinement of biomineralization models.

## Experiment and method

### Mollusk shell

The shells are sliced to approximately 1 mm in thickness from the edge of the shell. Selecting a sample from this location on the sagittal plane allowed the barrier between the nacre and prisms to be accessed. The sample was then epoxy-mounted onto pieces of silicon wafer, and ground and polished with diamond slurry progressively from a 5 to 1/8 μm grit size. Samples were polished at an oblique angle to the interlamellar organic layers to prevent interference of polishing scratches with our AFM images.

### IR s-SNOM

Due to its non-destructive nature, infrared vibrational nano-spectroscopy and -imaging were used to (1) chemically identify the organic phases, (2) identify the calcite polymorphs, and (3) map their spatial distributions prior to NanoSIMS analysis. Infrared vibrational nano-spectroscopy and -imaging was performed using IR *s*-SNOM as described previously (For additional technical details see Ref.^26^). In brief, broadband mid-infrared laser light is derived by optical parametric oscillator/difference frequency generation (OPO/DFG, Carmina, APE, Berlin, tunable from 3 to 15 µm). The light passes through an interferometer and is focused onto the metallic tip (160AC-GG, Opus) of an atomic force microscope (AFM) based IR *s*-SNOM setup (modified AFM+, Bruker). With the tip in tapping mode feedback with the sample surface, the tip-scattered near-field IR light is collected, detected with a mercury-cadmium-telluride detector (J15D12-M200-S050U-30-WE, Judson), and spectrally resolved by Fourier-transform interferometry. The detector signal is filtered using a lock-in amplifier (Zurich Instruments) at the second-harmonic of the tip tapping frequency for enhanced near-field signal discrimination. We further use narrowband pseudoheterodyne imaging for fixed wavelength imaging on selected resonances^27,28^.

We perform nano-FTIR IR *s*-SNOM which provides the vibrational, and thus chemically specific sample signature, with nanoscale spatial resolution. Through asymmetric interferometric near-field signal detection, we measure the real (Re_NF_) and imaginary (Im_NF_) part of the local vibrational sample response which corresponds to the underlying local values of n and k of the complex index of refraction $\widetilde{n}$^29,30^. By probing the amide I IR vibrational mode, we identify the relative protein content with sensitivity down to nanometer spatial scales^31–33^. Similarly, we distinguish mineral phases and their polymorphs based on their distinct vibrational spectral response^26^. The lower detector sensitivity and laser output power below < 1000 cm^−1^ required higher laser intensities in the reference arm and resulted in higher noise in the magnitude signal. Consequently, we display the phase of the IR *s*-SNOM signal ($\Phi$_NF_) which had lower noise than the Im_NF_ channel in this spectral range. For the localized molecular vibrational modes in this system, the *s*-SNOM phase $\Phi$_NF_ and imaginary part Im_NF_ have only minor peak position differences and both reflect the underlying sample complex index of refraction $\mathrm{Im}\left(\widetilde{n}\right)=k$^34^.

### NanoSIMS

For NanoSIMS imaging (NanoSIMS 50L, Cameca) samples were coated with 10 nm of high purity gold to improve conductivity. Analysis areas were pre-sputtered with 5 × 10^15^ Cs^+^ ions cm^−2^ at 16 keV beam energy. Subsequently, a ca. 1pA Cs^+^ beam with an estimated diameter of 100 nm was used to simultaneously collect ^12^C^−^, ^16^O^1^H^−^, ^12^C_2_^−^, ^12^C^14^N^−^, ^32^S–, and ^40^Ca^16^O^−^ ion images. 20 × 20 μm images, consisting of four frames each, were acquired at a resolution of 512 × 512 pixels, using a dwell time of 3375 μs px^−1^. Image frames were corrected for deadtime, integrated, and exported using the OpenMIMS plugin and the Fiji distribution of ImageJ^35^.

## Results and discussion

**Figure 2:**
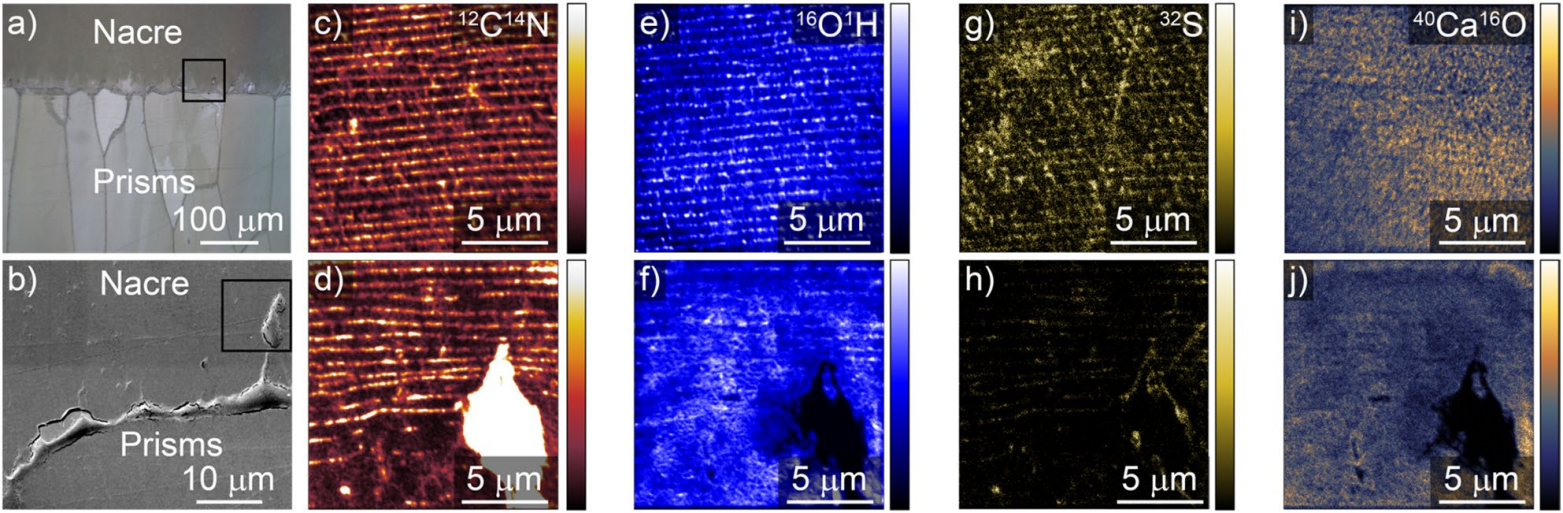
NanoSIMS mapping of boundary from mature nacre to FA. (**a**) Optical image of the interface between the nacre and the prismatic region. (**b**) SEM image of the region indicated by the square in (**a**) showing the prism-covering matrix, and its extension into the nacre. (**c**–**j**) NanoSIMS maps of ^12^C^14^N, ^16^O^1^H, ^32^S, and ^40^Ca^16^O of the nacre region. Images (**d**), (**f**), (**h**), and (**f**) correspond to the boxed region in b) and show interlamellar organic sheets in cross section parallel to the prism-covering membrane, with an extension of the prism-covering matrix in the lower right. Panels (**c**), (**e**), (**g**), (**i**) were acquired ~ 40 μm from the NPI in the mature nacre region.

Figure 2a shows an optical image of a representative region of interest at the NPI of *P. margaritifera.* The SEM image (panel b) shows the region near the NPI with an organic membrane separating the nacre and the prismatic region. As observed previously, we observe ~ 10–20 µm continuations of the prism-covering membrane extending into the nacre^14^. Figure 2c–j show corresponding NanoSIMS images of the nacre region for ^12^C^14^N, ^16^O^1^H, ^32^S, and ^40^Ca^16^O. The upper panels were acquired ~ 40 µm into the nacre in the orthogonal direction with respect to the NPI and the lower panels are acquired ~ 30 µm from the NPI where the prism-covering membrane extends into the nacre. The prism covering membrane shows high ^12^C^14^N signal indicative of organic nitrogen in the membrane. The interlamellar organic layers between the nacre tablets appear as high contrast horizontal lines in the NanoSIMS ^12^C^14^N map and with weaker contrast in the ^16^O^1^H and ^32^S map, consistent with prior studies^22^. The boundary between mature nacre and FA where the regular organic layers end is generally observed at the end of the prism-covering membrane continuations^14^. The ^40^Ca^16^O map shows a more uniform response outside of the dense organic region of the prism-covering membrane.

**Figure 3:**
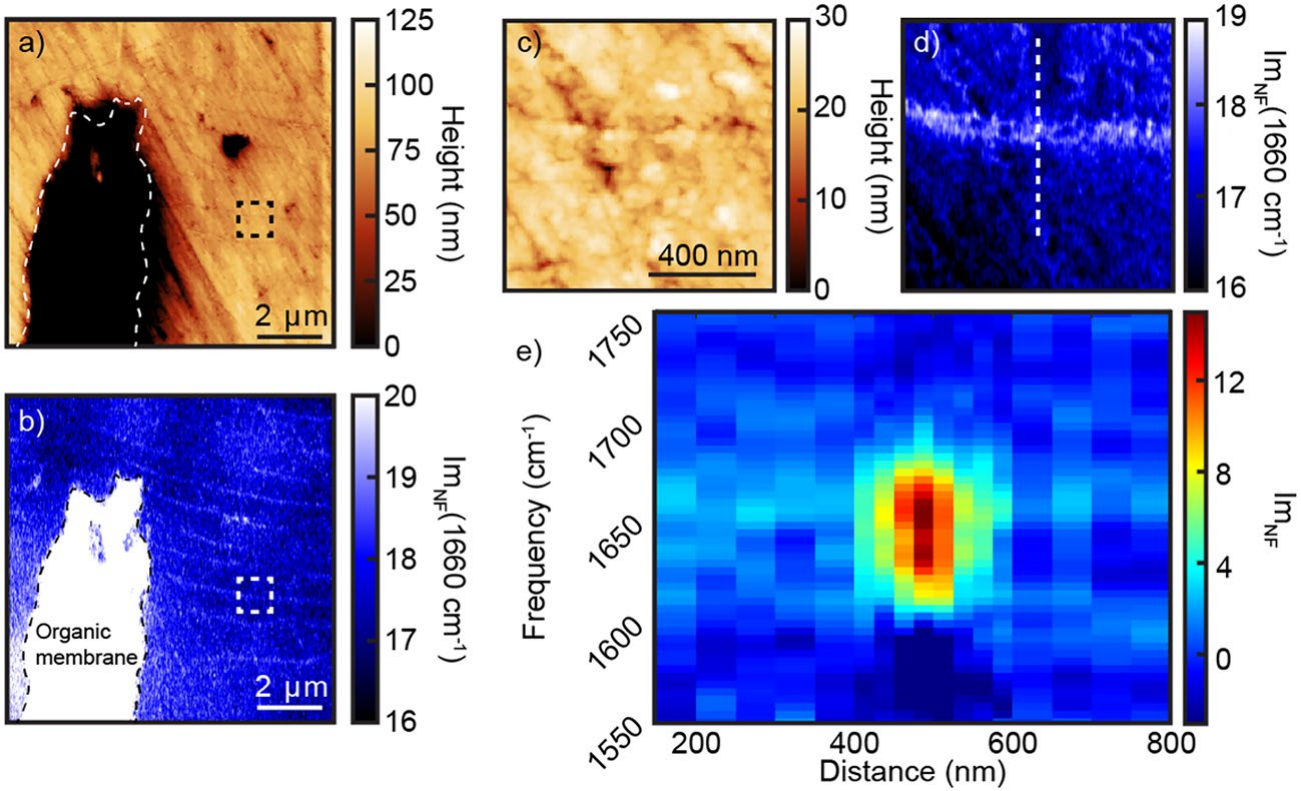
Spatio-spectral analysis of interlamellar organic layers in nacre (**a**) AFM height and (**b**) IR *s-*SNOM image at 1660 cm^-1^ mapping the amide I band in the nacre region. The prism-covering organic membrane extends ~ 30 μm into the nacre from the nacre/prismatic boundary. Near the end of the organic membrane, organic layers of ~ 50 nm in width appear with ~ 600 nm periodicity. (**c**) AFM topography and (**d**) IR *s-*SNOM of the subregion shown in (**a**) (black square). (**e**) Spatio-spectral transect along the dashed line in (**d**) demonstrating the amide I band with its spatial < 100 nm confinement in the interlamellar organic layer.

Figure 3a shows an AFM image at the end of a prism-covering membrane continuation in the nacre, which has a lower topography due to preferential removal of the organics during polishing. The corresponding IR *s*-SNOM map at 1660 cm^−1^ (b) shows high contrast within the prism-covering membrane, as well as the thin interlamellar organic layers which appear with ~ 600 nm periodicity^25^. Figure 3c, d shows the AFM and the IR *s-*SNOM Im_NF_, respectively, of the subregion indicated in Fig. 3a (black square) of the images. A spatio-spectral transect is shown in (e) acquired along the white dashed line in (d). The organic layer is characterized by a spectrally distinct and strong resonance at 1650 cm^−1^ corresponding to the amide I vibration of protein. The current model for the interlamellar organic membranes is a chitin sheet between two proteinaceous layers^19,36^. This model was developed through a combination of techniques, including coupling enzymatic treatment with scanning electron microscopy^25^, as well as cryo-transmission electron microscopy^19^. The protein layer width observed by IR *s-*SNOM of ~ 100 nm is consistent with prior electron microscopy observing width variations from 15 to 100 nm for these layers depending on the growth stage^25^.

**Figure 4:**
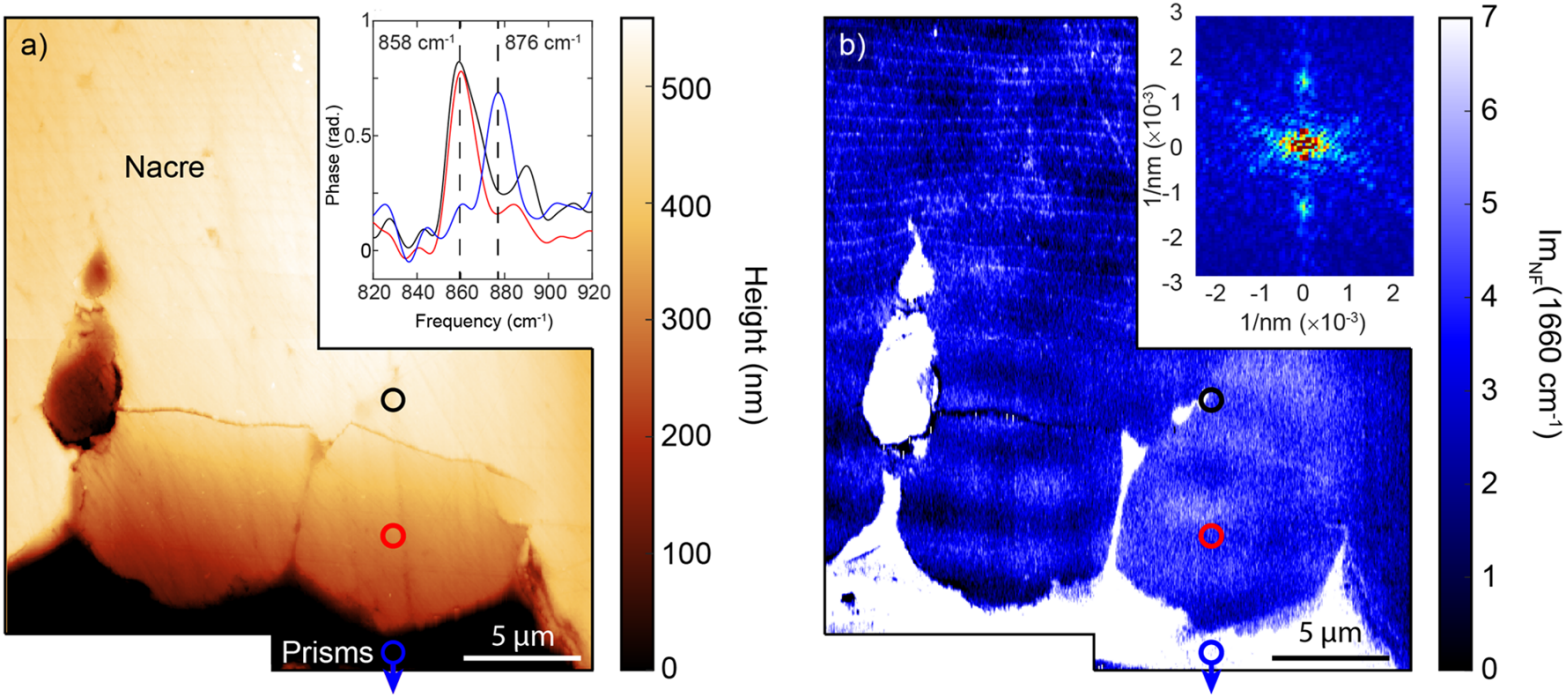
Organic mapping and minerology of nacre region. (**a**) AFM topography of boundary regions between the prismatic and the nacre regions. Two ~ 10 μm grains appear at the aragonite-calcite boundary. Inset: IR *s-*SNOM point spectra acquired at the locations indicated (colored circles). The nacre region (black circle) and the crystallites (red circle) are identified as aragonite by their characteristic peak at 858 cm^−1^, while the blue-shifted resonance at 876 cm^−1^ for the prismatic region (blue circle) is characteristic for calcite. (**b**) Corresponding amide I resonant IR *s*-SNOM image at 1660 cm^−1^ mapping the protein layers across this region. There are strong signatures near the boundary and extending outwards. Fainter striations begin near the end of the inclusion and extend into the nacre. The protein bands are periodic with ~ 600 nm spacing, as exemplified by the 2D FFT of the region shown in the inset.

We now seek to probe the relationship between proteins sheets and the calcium carbonate polymorph near the NPI and in the fibrous aragonite region. Figure 4 shows AFM (a) and IR *s-*SNOM (b) images near the NPI and a prism-covering membrane extension in the nacre. The IR *s-*SNOM image at 1660 cm^−1^ shows a strong signal on the organic membrane, as well as the thinner periodic interlamellar protein layers appearing ~ 15 μm into the mature nacre. A 2D FFT of the optical image shows the regular periodicity of the protein bands as sharp peaks located at (0, ± 1.5 × 10^3^ nm^−1^) corresponding to a periodicity of ~ 600 nm. Residing at the NPI are two micrometer-scale flat subregions separated by apparent enclosures of the prism covering membrane. We then perform IR *s-*SNOM spectroscopy to identify the calcium carbonate polymorphs across this section by analyzing the ${\nu }_{2}$ out-of-plane CO bending mode. In the mature nacre, we observe a peak at ~ 860 cm^−1^ corresponding to the aragonite polymorph, whereas in the prismatic region the peak is at ~ 876 cm^−1^, consistent with recent nano-IR spectroscopy of the polymorphs within a *M. edulis* shell^26^. In the fibrous aragonite region devoid of visible organic layers (Fig. 4, black spectrum in (a)), a peak position of ~ 860 cm^−1^ indicates that the fibrous aragonite region is still of the same polymorph as the mature nacre regions, as is the case within the two micrograins at the NPI (Fig. 4a, red spectrum). Identification of these subdomains as aragonite confirms that they form on the prism-covering membrane while the biomolecules specific to aragonite growth are present^37^. However, the organic membrane is in an apparently dynamic state at this stage with irregular growth patterns occasionally resulting in FA regions encapsulated within the prism-covering membrane.

**Figure 5:**
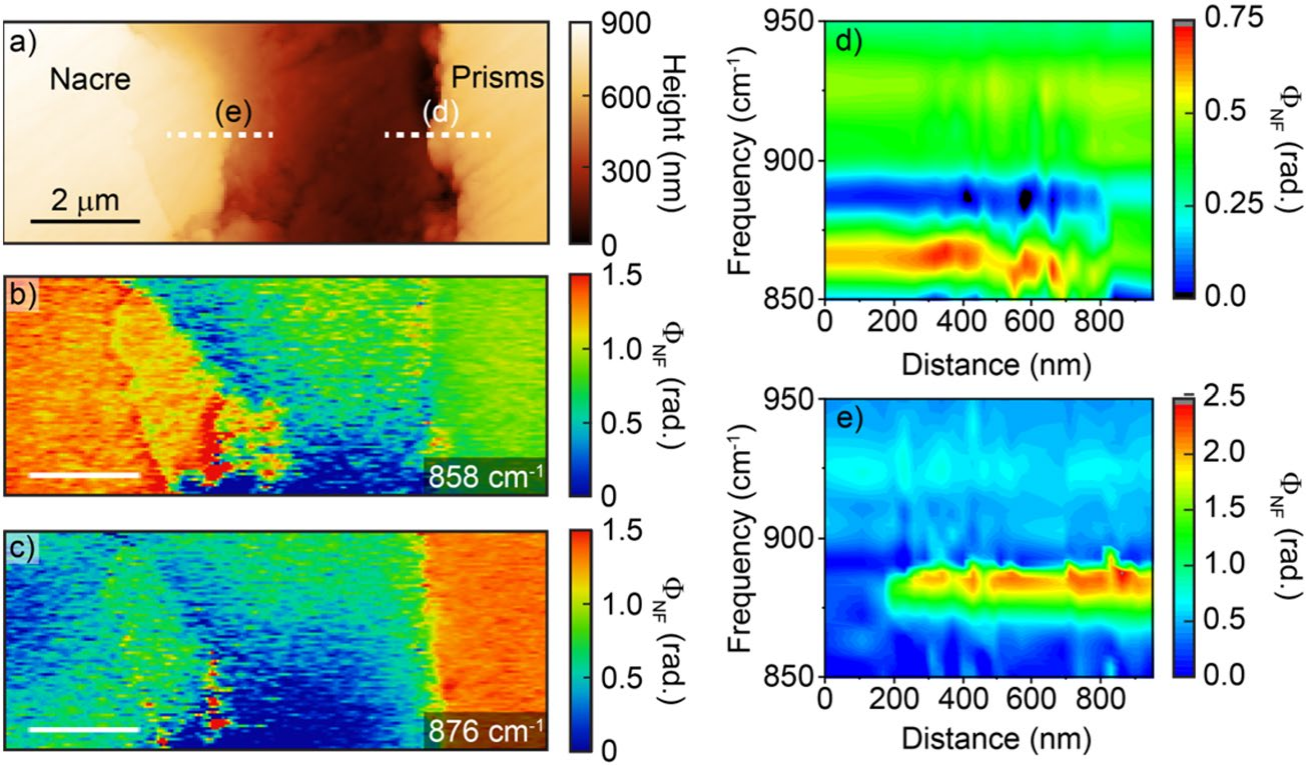
Minerology across the NPI. (**a**) Topography across the NPI, separated by the prism-covering membrane. *s*-SNOM images at (**b**) 860 cm^–1^ and (**c**) 880 cm^−1^ with contrast due to the distinct aragonite vs. calcite vibrational signatures. (**d**) Spatio-spectral linescan at the edge of the nacre shows the carbonite stretch at 860 cm^−1^, as well as an organic signature at 930 cm^−1^. (**e**) Spatio-spectral linecut along the prism edge with peak at 880 cm^−1^ indicative of calcite.

Figure 5 shows the spatio-spectral analysis of the minerology near the NPI. The AFM topography is shown in Fig. 5a and the corresponding IR *s*-SNOM images acquired at 858 cm^−1^ (Fig. 5b) and 876 cm^−1^ (Fig. 5c) show contrast at their respective calcite and aragonite vibrational frequencies. We then performed spatio-spectral linecuts across the edge of these boundaries to identify changes in polymorph across these regions. The linecut for the aragonite edge is shown in Fig. 5d. The ${\nu }_{2}$ peak is visible and decreases in intensity as we move into the organic layer. The organic band from 910 to 940 cm^−1^ present in the prism-covering organic membrane is also present in the spectra of the FA region mapped in panel e, indicating organics are mixed with the nacre in the first few hundred nanometer layer. The spatio-spectral linecut across the calcite boundary is shown in Fig. 5e, and shows a consistent ${\nu }_{2}$ response at 880 cm^−1^. There has been debate as to the existence of amorphous structures in the incipient calcite prisms, as evidenced by electron diffraction studies^38^, transmission electron microscopy^24^, and coherent and stimulated Raman spectroscopy^39,40^. Our results do not show the presence of an amorphous phase above a 100 nm scale from the NPI in the calcite region, which would be evidenced by a broadening of ${\nu }_{2}$ and its shift to 874 cm^−1^^41^. The slight red-shift and variable intensity of the peak over the first few hundred nanometers of the FA could indicate a thin amorphous region and an increased incorporation of organics at the interface between the FA and the prism covering membrane. Electron microscopy studies have shown evidence of few nanometer organic layers between ~ 30–50 nm fibrous aragonite granules^22^. With the size of these granules and the organic layers below our spatial resolution of ~ 50 nm, an increase in the thickness and density of these organic layers would not be directly resolved, but would show up as a decrease in the carbonate peak intensity as we observe in Fig. 5d.

**Figure 6:**
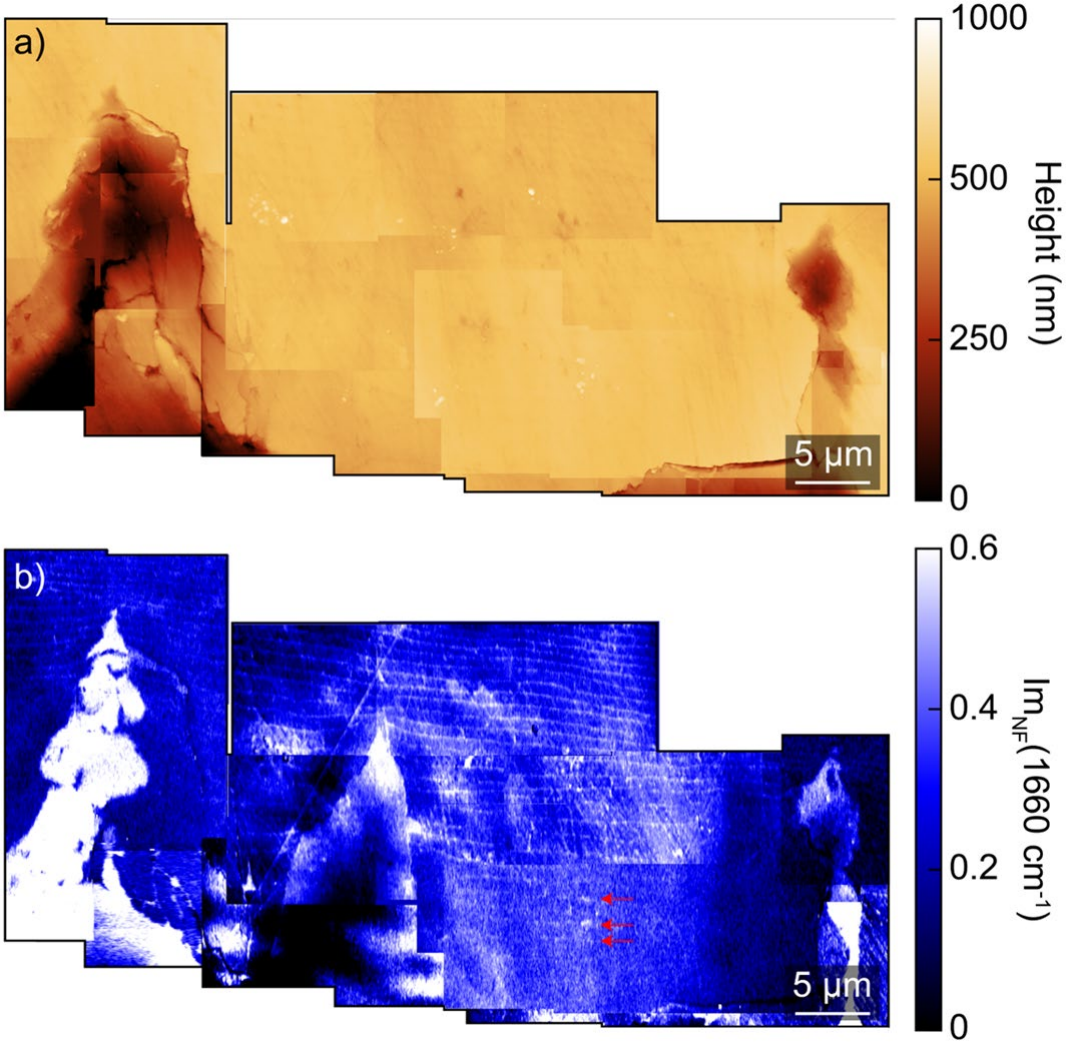
Region with organic pattern and nacre tablet morphology. (**a**) AFM topography and (**b**) corresponding IR *s*-SNOM image acquired at 1660 cm^−1^ mapping the protein sheets across a ~ 60 μm region showing long range order. The regular periodic striations appear ~ 15 μm from the NPI to the left and right of the prism-covering membrane inclusions. However, the nacre tablets extend down to within ~ 5 μm of the NPI boundary in the center of the image, with decreasing regularity and contrast due to increased dispersed organics in the carbonate (red lines).

Figure 6 shows AFM (a) and corresponding IR *s*-SNOM (b) image at 1660 cm^−1^ across a ~ 60 μm region in the vicinity of two prism-covering membrane extensions with dense organic IR *s*-SNOM signatures. This multiscale map reveals both long range order and larger scale disorder with nanoscale resolution. It was obtained by combining several smaller scale images by locating common topographic features as fiducial markers in adjacent images. The relatively flat region between the two inclusions exhibits notable deviations from the previously observed patterns noted in Figs. 2 and 3. While the amide striations to the right and left of the two prism-covering membrane extensions end 15–20 μm from the NPI, they appear to extend down to ~ 5 μm from the NPI in the center of the image. In this region, the lines are more irregular and less well-defined due in part to an increased amide background signal which could be associated with dispersed organics in this disordered region. Cutting through these irregular lines is a triangular organic-rich region with no regular striation. This is an area where the tablet growth is not well controlled and are not fully formed, and organics are mixed with the mineral.

**Figure 7:**
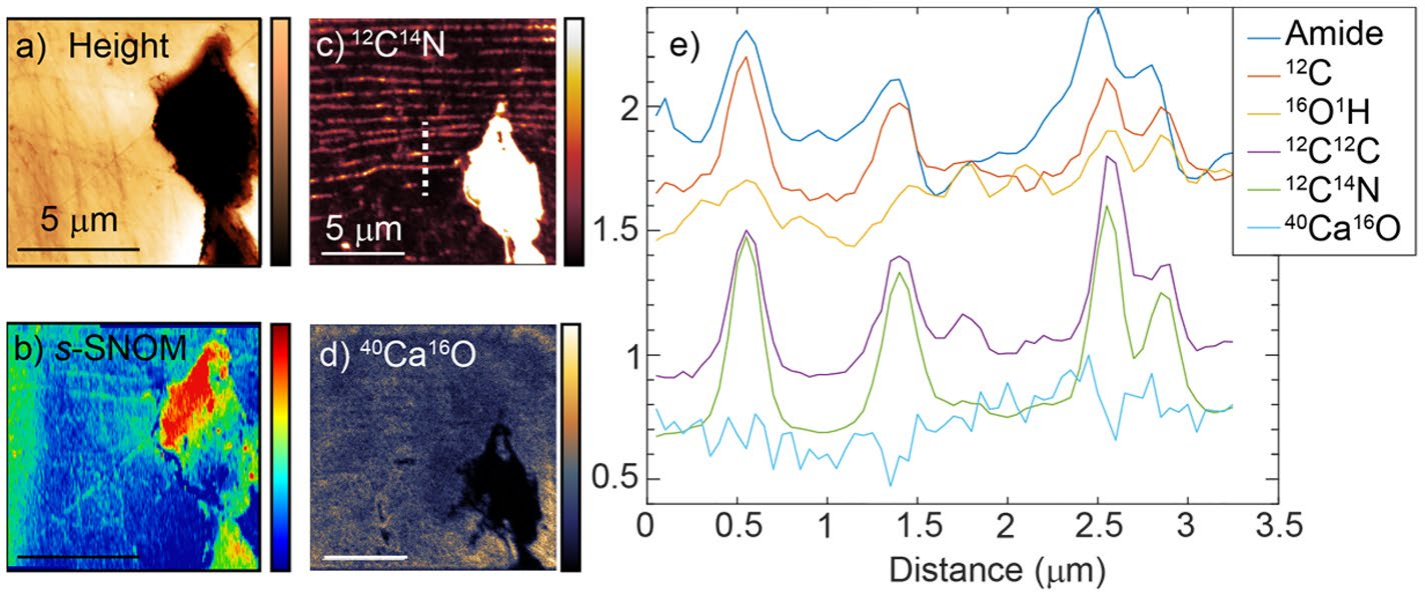
Correlated IR *s*-SNOM, and NanoSIMS of nacre region. (**a**) The AFM height and (**b**) *s*-SNOM phase map at 1660 cm^−1^ of the terminus of a prism-covering matrix extension. Correlated NanoSIMS correlates the presence of (**c**) ^12^C^14^N and (**d**) ^40^Ca^16^O. (**j**) A correlated linecut of the *s*-SNOM and NanoSIMS images (white dashed line in (**c**)) is displayed in (**e**) and shows the correlation between the amide-I response and ^12^C^14^N, ^12^C^12^C, and ^12^C.

Figure 7 shows NanoSIMS and *s*-SNOM analyses across the same region allowing correlation of the elemental signature with chemical composition. First, AFM (panel a) and *s*-SNOM (panel b) imaging was performed to image the spatial distribution of amide I across a region of nacre. This was performed near the boundary of the mature and FA, at the end of a prism covering membrane extensions in the nacre. We see the thin inter-tablet organic layers in the amide I IR *s-*SNOM map. We then performed NanoSIMS (Fig. 7c,d) in the same region, targeting organic elemental and molecular signatures. A linecut along the white dashed line in the CN map is shown in panel e). The amide *s*-SNOM signature (blue) is well-correlated with the ^12^C, ^12^C^14^N, and the ^12^C^12^C NanoSIMS signals, consistent with the organic carbon within these striations being proteinaceous.

## Summary

The results show the combined utility of correlated IR *s*-SNOM and NanoSIMS imaging to investigate biomineralization processes through nanoscale chemical and elemental imaging in *P. margaritifera*. Multiscale mapping of organics tracks the morphological variations of the nacre structure across 10’s of μm field of view with 10’s of nanometer spatial resolution. IR *s*-SNOM imaging and spectroscopy identifies the organic layers observed in NanoSIMS as protein with ~ 600 nm periodicity and ~ 100 nm confinement. While IR *s*-SNOM confirms that the nacre consists of aragonite down to within 300 nm of the NPI, at closer distances it is mixed with organic with a less well-defined crystal structure. The morphology of the nacre varies from the mature nacre tablets with regular arrangement, to the disordered FA region near the NPI, as well as the encapsulated granules residing along the prism-covering membrane in the incipient layer of nacre. The latter indicates that the organic membrane is in a dynamic state during the deposition of the nacre. Combined with NanoSIMS elemental maps IR *s*-SNOM with simultaneous nanoscale spatial resolution across mesoscale region of interests, can capture key moments in biomineralization and correlate nanoscale polymorph changes with organic distribution and yield mechanistic information about these processes.

## Data Availability

The datasets used and/or analysed during the current study available from the corresponding author on reasonable request.
