# Publisher Correction: Correlative chemical and elemental nano-imaging of morphology and disorder at the nacre-prismatic region interface in *Pinctada margaritifera*

**DOI:** 10.1038/s41598-023-50290-2

**Published:** 2024-01-08

**Authors:** Brian T. O’Callahan, Amy Larsen, Sarah Leichty, John Cliff, Alex C. Gagnon, Markus B. Raschke

**Affiliations:** 1 Environmental and Molecular Sciences Division, Pacifc Northwest National Laboratory, Richland, WA USA; 2 School of Oceanography, University of Washington, Seattle, WA USA; 3 Department of Physics, and JILA, University of Colorado at Boulder, Boulder, CO USA

Correction to: *Scientifc Reports* 10.1038/s41598-023-47446-5, published online 01 December 2023.

The original version of this Article contained an error in the copyright line.

“© The Author(s) 2023”

now reads:

“© Battelle Memorial Institute, Amy Larsen, Alexander C. Gagnon, Markus B. Raschke 2023”

The original Article has been corrected.

